# Availability, prices, and affordability of selected essential cancer medicines in a middle-income country – the case of Mexico

**DOI:** 10.1186/s12913-020-05167-9

**Published:** 2020-05-14

**Authors:** Daniela Moye-Holz, Margaret Ewen, Anahi Dreser, Sergio Bautista-Arredondo, Rene Soria-Saucedo, Jitse P. van Dijk, Sijmen A. Reijneveld, Hans V. Hogerzeil

**Affiliations:** 1 Department of Community and Occupational Medicine, University Medical Center Groningen, University of Groningen, Hanzeplein 1, 9713 GZ Groningen, The Netherlands; 2 Health Action International (HAI), Overtoom 60, 1054 HK Amsterdam, the Netherlands; 3 National Institute of Public Health (INSP), Avenida Universidad 655, Santa María Ahuacatitlán, 62100 Cuernavaca, Morelos Mexico; 4 Boston University School of Public Health, 715 Albany St, Boston, Massachusetts 02118, EE. UU USA

**Keywords:** Availability, Affordability, Prices, Essential cancer medicines, Mexico

## Abstract

**Background:**

More alternatives have become available for the diagnosis and treatment of cancer in low- and middle-income countries. Because of increasing demands, governments are now facing a problem of limited affordability and availability of essential cancer medicines. Yet, precise information about the access to these medicines is limited, and the methodology is not very well developed. We assessed the availability and affordability of essential cancer medicines in Mexico, and compared their prices against those in other countries of the region.

**Methods:**

We surveyed 21 public hospitals and 19 private pharmacies in 8 states of Mexico. Data were collected on the availability and prices of 49 essential cancer medicines. Prices were compared against those in Chile, Peru, Brazil, Colombia and PAHO’s Strategic Fund.

**Results:**

Of the various medicines, mean availability in public and private sector outlets was 61.2 and 67.5%, respectively. In the public sector, medicines covered by the public health insurance “People’s Health Insurance” were more available. Only seven (public sector) and five (private sector) out of the 49 medicines were considered affordable. Public sector procurement prices were 41% lower than in other countries of the region.

**Conclusions:**

The availability of essential cancer medicines, in the public and private sector, falls below World Health Organization’s 80% target. The affordability remains suboptimal as well. A national health insurance scheme could serve as a mechanism to improve access to cancer medicines in the public sector. Comprehensive pricing policies are warranted to improve the affordability of cancer medicines in the private sector.

## Background

Comprehensive cancer care requires a number of interventions, from specialized diagnostics to various treatments including: surgery, radiotherapy, and chemotherapy [1, 2]. Low-cost and effective medicines to treat several cancers exist in generic form [3]. However, the prices of many cancer medicines (both generic and originators) make them unaffordable for governments and patients, and contribute to their in health facilities in many low- and middle-income countries (LMIC) [4]. Unavailable and unaffordable essential cancer medicines may lead to substandard and/or interrupted treatment regimens, worse health outcomes, and lower chances of survival [2]. Therefore, equitable access to affordable essential medicines is a crucial component of comprehensive cancer care [3, 5–7].

In absolute terms, cancer has become a leading cause of death and disability around the globe. Yet, many patients in LMIC remain untreated [2, 3, 8, 9], and access to cancer care, including medicines, is becoming a priority. Describing the current access to cancer medicines and understanding the barriers that hinder their accessibility [10] are key components to develop responsive national policies, and to measure their impact. In general, comparable information about access to cancer medicines is limited worldwide [10, 11]. Direct assessment of the availability and affordability of essential cancer medicines has rarely been conducted in LMICs [11], except in Tanzania [12] and Pakistan [13]. Most studies have been limited to infectious diseases and medicines to treat non-communicable diseases (NCDs) other than cancer [14–17].

In Mexico, cancer accounts for nearly 13% of deaths [18–20]. Cancer care in Mexico is available in the public and private sectors. In the public sector, social health insurance institutions provide comprehensive health services (including cancer treatment) to employees in the formal sector. People who are ineligible for social health insurance can affiliate to the People’s Health Insurance (Seguro Popular de Salud - SPS) – a federal government insurance scheme that reimburses health facilities (usually Ministry of Health (MoH) facilities) according to a catalog of services [21, 22]. This population can also receive coverage for high-cost services (e.g. all pediatric cancers and eight types of cancers in adults) through the Fund against Catastrophic Expenditures (FPGC) [2, 23]. SPS patients receive healthcare and medicines at the point of service with no additional cost. For those diseases not covered by SPS, patients might pay out-of-pocket for some services and medicines according to their level of income. The private sector consists of private pharmacies and health facilities where patients pay additional insurance contributions or out-of-pocket [21]. While several studies have investigated insurance coverage for breast, cervical and children cancer care in the public sector [23–25], there is scant research on the availability and affordability of cancer medicines.

The purpose of this study was to assess and compare the availability and affordability of essential cancer medicines in the public and private sectors in Mexico. We also compared consumer procurement and consumer prices in the two sectors against those in four other middle-income countries in the region (Brazil, Colombia, Chile and Peru).

## Methods

The standardized World Health Organization/Health Action International (WHO/HAI) methodology [26] that measures medicine prices, availability and affordability was adapted to study essential medicines for cancer, collecting data from a sample of public hospitals and private pharmacies across the country.

### Sample

We selected 49 cancer medicines (each strength and dose-form specific) from the national formulary. According to the national clinical guidelines, the SPS protocols and the National Institute of Cancerology’s (INCAN) guidelines, these medicines are required for the treatment of breast cancer, colorectal cancer, leukemia and renal cancer. Of these, the SPS reimburses 40 medicines (for more information on the medicines of study see Additional file 1).

Data were collected in Mexican states where cancer care is provided. A total of 26 specialized and tertiary hospitals in the public sector were selected from eight of 32 states of the country, with differing levels of marginalization [27, 28], Organization for Economic Co-operation and Development (OECD) health well-being levels [29], and with at least one MoH hospital specialized in cancer care (Table 1). In this way, our sample captured the variability and characteristics of the health system. We surveyed all MoH hospitals providing cancer care in the selected states except in Chihuahua, Oaxaca and Veracruz. We did not survey five hospitals in these three states due to logistical issues and hospital’s refusal to participate.

**Table 1:** Characteristics of selected states and number of facilities surveyed

State	Level of Marginalization	OECD^a^ Health well-being indicator	No. Public Hospitals surveyed	No. Private Pharmacies surveyed
Campeche	High	Low	1	1
Oaxaca	High	Low	2	2
Veracruz	High	Low	3	2
Chihuahua	Medium	Low	2	2
Guanajuato	Medium	Medium	3	3
Yucatan	Medium	Medium	2	3
Jalisco	Low	High	4	3
Mexico City	Low	High	4	3

^a^*OECD* Organization for Economic Co-operation and Development

Cancer medicines are not always continuously stocked in private pharmacies. Instead, pharmacies that dispense these medicines usually do so from a fixed inventory list, with next-day delivery. We selected 1 to 3 pharmacies that market specialty medicines (e.g. cancer medicines) in each of the eight states, resulting in a total of 19 pharmacies.

### Data collection

The survey was conducted from March to June 2017. From the public sector hospital pharmacies, we recorded whether the medicine was in stock at the time of the visit (yes/no), and the price paid by the hospital and by the patient (if applicable). We did not distinguish between originator brands or generics, because the Mexican public sector regularly procures generic versions of multisource medicines and only provides originator (patented) medicines when no generics are available.

In the private sector pharmacies, we recorded whether the medicine was included in the pharmacy’s inventory and could be ordered for next-day delivery (yes/no), the price paid by patients of the lowest-priced generic available and the price of the patented medicines.

### Data analysis

We conducted the data analysis using the WHO/HAI methodology workbook [26] as described in the following sections.

#### Availability

We assessed availability in each sector by calculating the percentage of facilities where each medicine was available, and the mean across a collection of medicines. In the public sector, we also compared availability per disease, since breast cancer and colorectal cancer are covered by SPS for adults and children, while leukemia and renal cancer are covered for children only.

#### Affordability

We assessed affordability from the patient’s perspective when paying out-of-pocket. For each medicine, we compared the defined daily dose values [30] and the median unit price with the minimum daily wage in 2017 [26, 31]. Based on affordability assessments by Khatib et. Al [32] and Sarwar et al. [13], we considered a medicine as affordable if 20% or less of the daily wage was needed to pay for 1 day of medicine [13, 32]. In public hospitals, affordability was only assessed in the three hospitals where patients pay out-of-pocket for cancer medicines. We were not able to include data from the other hospitals, as these reported that patients did not pay out-of-pocket for their medicines or only paid a co-payment (for all healthcare services) according to their level of income when they do not have SPS coverage.

#### Prices and international price comparison

The WHO/HAI methodology expresses prices as a ratio (Median Price Ratio (MPR)) to international reference prices, which are the median supplier prices reported by Management Sciences for Health (MSH) [33]. As MSH reported prices for a limited number of the study medicines, prices from publicly accessible websites of comparable countries in the region were also used as external benchmark, namely Brazil, Colombia, Chile (recently considered a high-income country) and Peru [34–37]. To calculate MPRs, MSH international reference prices, the Pan-American Health Organization (PAHO) Strategic Fund procurement prices and 2017 median public sector procurement prices in the comparator countries were compared with Mexican public procurement prices. For patient prices, comparisons were made with median patient prices of patented and lowest priced generic medicines in the comparator countries.

## Results

### Availability and affordability of medicines in the public and private sectors

Table 2 shows the availability and affordability of cancer medicines in the public hospitals and private pharmacies we surveyed. In the public sector, the overall mean availability was 61.2%, with 70.2% availability of the SPS medicines. Mean availability of medicines was: for breast cancer 81.8%, for leukemia 69.2%, for colorectal cancer 62.9%, and renal cancer 57.3%. Overall availability in the private sector was 67.4%. Generic medicines (60.6%) were more available than patented medicines (54.0%). The availability of patented medicines was below 50% in the public and private sectors for most medicines except bevacizumab 400 mg, L-asparaginase, mercaptopurine, rituximab 100 mg and 500 mg, and trastuzumab.

**Table 2:** Availability and affordability of 49 selected cancer medicines in public hospitals and private pharmacies

		Availability		Affordability^**a**^	
**No.**	**Medicine**	**Public Sector (*n*** **= 21)**	**Private Sector (*n*** **= 19)**	**Public Sector (n = 21)**	**Private Sector**^**b**^**(n = 19)**
1	Anastrozole 1 mg tab	76.19%	89.47%	0.24	0.59
2	Bevacizumab 100 mg inj	42.86%	73.68%	30.12	57.68
3	Bevacizumab 400 mg inj	66.67%	68.42%	29.42	51.99
4	Capecitabine 500 mg tab	76.19%	78.95%	1.88	5.50
5	Carboplatin 150 mg inj	85.71%	68.42%	0.44	0.83
6	Cetuximab 5 mg/ml inj	38.10%	36.84%	35.74	62.88
7	Cyclophosphamide 200 mg inj	80.95%	84.21%	0.48	0.67
8	Cyclophosphamide 500 mg inj	95.24%	73.68%	0.53	0.64
9	Cytarabine 500 mg inj	95.24%	84.21%		
10	Dasatinib 50 mg tab	4.76%	31.58%	17.20	33.95
11	Daunorubicin 20 mg inj	66.67%	63.16%		
12	Docetaxel 20 mg/1 ml inj	66.67%	78.95%	5.26	8.00
13	Docetaxel 80 mg/4 ml inj	71.43%	84.21%	3.78	6.59
14	Doxorubicin 10 mg inj	85.71%	73.68%	0.61	1.09
15	Doxorubicin 50 mg inj	85.71%	84.21%	0.45	0.57
16	Epirubicin 10 mg/5 ml inj	42.86%	63.16%		3.00
17	Epirubicin 50 mg/25 ml inj	61.90%	84.21%	0.57	1.86
18	Etoposide 20 mg/ml inj	95.24%	78.95%	0.16	0.33
19	Everolimus 10 mg tab	14.29%	42.11%	21.26	30.17
20	Everolimus 5 mg tab	9.52%	42.11%		30.17
21	Exemestane 25 mg tab	85.71%	78.95%	1.02	0.73
22	Fluorouracil 250 mg inj	80.95%	52.63%	0.10	0.16
23	Folinic Acid 50 mg/4 ml inj	95.24%	63.16%		
24	Folinic Acid 15 mg tab	42.86%	57.89%		
25	Gemcitabine 1 g inj	76.19%	78.95%	1.09	4.86
26	Ifosfamide 1 g inj	85.71%	73.68%	3.15	5.50
27	Imatinib 100 mg tab	33.33%	63.16%	3.58	17.59
28	Imatinib 400 mg tab	38.10%	47.37%	3.43	18.63
29	Irinotecan 20 mg/ml inj	76.19%	68.42%	3.18	7.25
30	L-Asparaginase 10,000 IU inj	76.19%	84.21%	13,15	17.03
31	Letrozole 2.5 mg tab	66.67%	84.21%	0.20	0.44
32	Mercaptopurine 50 mg tab	52.38%	84.21%	1.45	2.38
33	Methotrexate 2.5 mg tab	71.43%	84.21%	0.02	0.07
34	Methotrexate 500 mg inj	80.95%	78.95%	0.02	0.02
35	Methotrexate 50 mg inj	95.24%	68.42%	0.06	0.11
36	Nilotinib 200 mg tab	23.81%	36.84%		21.72
37	Oxaliplatin 100 mg/20 ml inj	61.90%	84.21%	0.89	2.36
38	Oxaliplatin 50 mg/10 ml inj	57.14%	73.68%	1.25	2.40
39	Paclitaxel 6 mg/ml inj	80.95%	78.95%	0.45	1.63
40	Panitimumab 20 mg/ml inj	28.57%	42.11%	31.72	58.93
41	Pazopanib 200 mg tab	9.52%	26.32%		14.89
42	Pazopanib 400 mg tab	4.76%	52.63%	8.49	14.89
43	Rituximab 100 mg/10 ml inj	61.90%	68.42%	6.65	30.97
44	Rituximab 500 mg/50 ml inj	71.43%	68.42%	4.65	30.96
45	Sorafenib 200 mg tab	19.05%	57.89%	19.59	31.13
46	Sunitinib 12.5 mg tab	14.29%	47.37%	11.89	25.10
47	Tamoxifen 20 mg tab	90.48%	78.95%	0.02	0.06
48	Trastuzumab 440 mg inj	71.40%	57.89%	13.77	23.63
49	Vincristine 1 mg inj	85.71%	78.95%	0.35	0.45
	**Mean availability/Median affordability**	61.20%	67.45%	1.45	5.50
	**Mean availability/Median affordability:**				
	**Breast cancer medicines**	74.8%	78.1%	0.57	1.36
	**Leukemia medicines**	59.3%	66.4%	3.50	17.72
	**Colorectal cancer medicines**	55.6%	58.3%	2.53	6.37
	**Renal cancer medicines**	52.0%	61.9%	1.09	17.72

*tab* tablet, *inj* injectable, *mg* milligrams, *ml* milliliters, *IU* international unit, *n* number of facilities surveyed and included in the analysis

^a^Affordability is expressed as number of days needed to purchase 1 day of treatment based in minimum daily wage

^b^Affordability in the private sector: we present only the affordability of the lowest priced generic medicines. For medicines with no generic alternatives, we present the affordability of the patented/originator medicine

In the public sector, the median affordability (*n* = 49 medicines) was 1.45 days’ wages required to purchase 1 day’s supply. Seven medicine products were considered affordable, that is, 1 day of medicine supply costs 20% or less of the daily wage: etoposide, fluorouracil, letrozole, methotrexate (2.5 mg tablet (tab), 500 mg injectable (inj) and 50 mg inj) and tamoxifen. In the private sector, five medicine products were considered affordable: fluorouracil, methotrexate 500 mg inj and 50 mg inj, tamoxifen, and methotrexate 2.5 mg tab. The median price of overall medicines in the private sector was 5.50 days’ wages needed to buy 1 day of one medicine’s supply. The median affordability of patented medicines was 30.17 days’ wages needed to buy 1 day of one medicine’s supply. For the generic medicines, it was 0.78 days’ wages.

### Procurement and patient prices

**Fig. 1:**
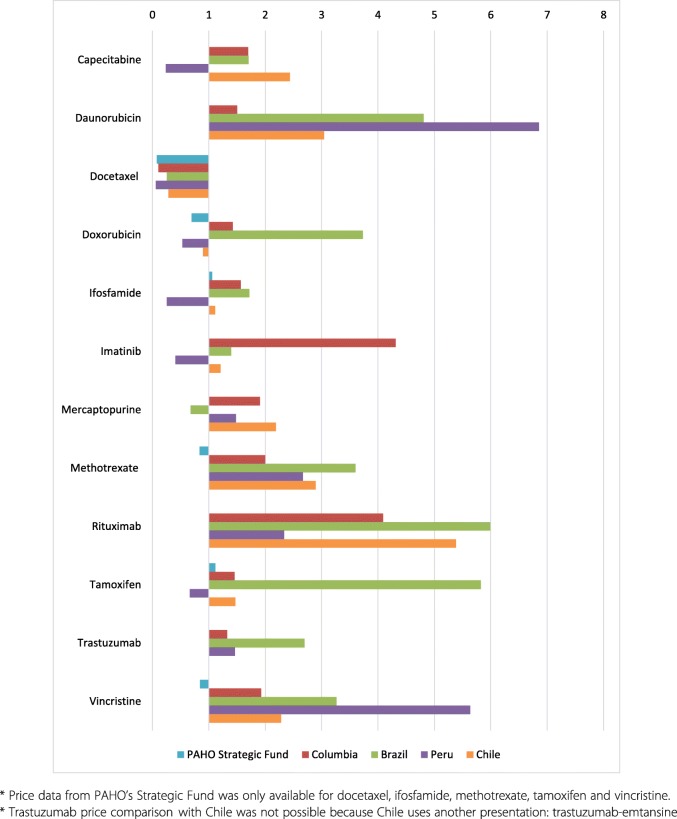
Price differences between public procurement prices in Mexico (=1) and other LATAM countries and PAHO’s Strategic Fund

Table 3 shows median public sector procurement prices and median patient prices in the private sector, both in local currency (Mexican peso (Mex$)) and as ratios to median prices in the four comparator countries and international reference prices. In the public sector, the overall median procurement price in Mexico was 0.59 times (41% below) the median comparator country price and 0.80 times (20% below) the MSH international reference prices. However, the prices of a few individual medicines were over twice the median comparator country price, e.g. MPR of anastrozole (2.12x), docetaxel 80 mg (5.56x) and 20 mg inj (3.40x). The prices of docetaxel 20 mg/ml (12.42x) and 80 mg/ml (12.08x), folinic acid 50 mg/ml (3.88x) and irinotecan (6.16x) were over three times the MSH international reference prices. For patient prices in the private sector, overall medicines had median MPRs of 0.65 (35% below the reference prices). Cancer medicines ranged from an MPR of 0.13 for oxaliplatin 50 mg inj to 2.48 for docetaxel 80 mg inj. Most medicine prices were 2 to 6 times higher in the other Latin American (LATAM) countries compared to Mexico. On the other hand, six medicines were cheaper in Peru. Only the price of docetaxel was consistently higher in Mexico (Fig. 1).

**Table 3:** Median public sector procurement prices and median patient prices, in local currency and as price ratios to median prices in the four-comparator countries

		Public Sector Procurement Price			Private Sector Patient Price^**a**^	
No.	Medicine Name	Median Price (Mex$)	MPR (LATAMc)	MPR (MSH)	Median Price (Mex$)	MPR (LATAMc)
1	Anastrozole 1 mg tab	24.29	2.12	2.35	47.36	1.27
2	Bevacizumab 100 mg inj	53.58	0.58		102.59	0.82
3	Bevacizumab 400 mg inj	48.67	0.66		92.48	0.84
4	Capecitabine 500 mg tab	22.07	0.59	0.67	73.33	0.89
5	Carboplatin 150 mg inj	1.27	0.31	0.65	2.67	0.21
6	Cetuximab 5 mg/ml inj	40.81	0.61		77.43	1.01
7	Cyclophosphamide 200 mg inj	0.15	0.41	0.36	0.21	0.35
8	Cyclophosphamide 500 mg inj	0.14	0.37	0.45	0.20	0.42
9	Cytarabine 500 mg inj	0.30	1.08	1.66	0.60	0.58
10	Dasatinib 50 mg tab	573.78	0.57		1132.27	1.46
11	Daunorubicin 20 mg inj	5.90	0.25	0.31	12.99	0.31
12	Docetaxel 20 mg/1 ml inj	62.93	3.40	12.42	99.53	0.54
13	Docetaxel 80 mg/4 ml inj	51.83	5.56	12.08	82.00	2.48
14	Doxorubicin 10 mg inj	7.45	1.23	1.79	17.53	0.42
15	Doxorubicin 50 mg inj	3.92	0.86	1.38	9.20	0.49
16	Epirubicin 10 mg/5 ml inj	12.84	0.36	1.20	34.35	0.59
17	Epirubicin 50 mg/25 ml inj	6.20	0.41	0.73	21.28	0.38
18	Etoposide 20 mg/ml inj	0.49	0.64	1.30	1.06	2.01
19	Everolimus 10 mg tab	1702.00	0.83		2414.97	0.65
20	Everolimus 5 mg tab	425.50	0.44		1207.48	0.65
21	Exemestane 25 mg tab	69.18	1.08	1.69	58.50	0.66
22	Fluorouracil 250 mg inj	0.08	0.49	0.38	0.13	0.63
23	Folinic Acid 50 mg/4 ml inj	3.56	0.76	3.88	4.60	1.14
24	Folinic Acid 15 mg tab	15.84	0.71	0.62	23.33	0.80
25	Gemcitabine 1 g inj	0.44	0.32	0.89	1.95	0.37
26	Ifosfamide 1 g inj	0.27	0.75	1.30	0.63	0.83
27	Imatinib 100 mg tab	57.32	0.54	4.22	281.58	0.52
28	Imatinib 400 mg tab	219.48	0.77	0.44	1192.74	0.66
29	Irinotecan 20 mg/ml inj	6.97	1.20	6.16	19.34	0.35
30	L-Asparaginase 10,000 IU inj	0.11	0.49	1.06	0.14	0.92
31	Letrozole 2.5 mg tab	2.62	0.04	0.28	35.00	1.03
32	Mercaptopurine 50 mg tab	33.24	0.59	0.76	54.48	1.25
33	Methotrexate 2.5 mg tab	1.20	0.36	0.39	5.81	1.73
34	Methotrexate 500 mg inj	0.45	1.13	1.35	0.79	0.61
35	Methotrexate 50 mg inj	1.22	0.59	0.59	3.44	1.05
36	Nilotinib 200 mg tab	279.07	0.56	11.92	579.59	0.84
37	Oxaliplatin 100 mg/20 ml inj	6.36	0.78	0.43	17.15	0.18
38	Oxaliplatin 50 mg/10 ml inj	8.52	1.11	0.75	17.45	0.13
39	Paclitaxel 6 mg/ml inj	2.78	0.76		8.72	0.58
40	Panitimumab 20 mg/ml inj	76.99	0.84		157.23	1.18
41	Pazopanib 200 mg tab	170.44	0.56		297.90	0.59
42	Pazopanib 400 mg tab	339.86	0.57		595.85	0.45
43	Rituximab 100 mg/10 ml inj	13.09	0.45	0.49	77.46	0.85
44	Rituximab 500 mg/50 ml inj	10.82	0.21	0.31	77.44	1.26
45	Sorafenib 200 mg tab	404.04	0.73		622.82	0.67
46	Sunitinib 12.5 mg tab	326.01	0.43		717.58	0.53
47	Tamoxifen 20 mg tab	1.46	0.68	0.66	4.90	0.41
48	Trastuzumab 440 mg inj	49.29	0.68		94.56	0.97
49	Vincristine 1 mg inj	53.66	0.36	0.84	101.00	0.53
	**Median**	12.84	0.59	0.80	41.18	0.65

*OB* originator brand, *LPG* lowest price generic, *tab* tablet, *inj* injectable, *mg* milligrams, *ml* milliliters, *IU* international unit, *n* number of facilities surveyed and included in the analysis, *LATAMc* Latin American countries, *MPR* median price ratio, *Mex$* mexican peso, *MSH* management sciences for health

^a^Median prices and MPR in the private sector: we present only the median prices and the MPR of the lowest priced generic medicines. For medicines with no generic alternatives, we present the median prices and the MPR of the patented/originator medicine

## Discussion

In Mexico, the overall availability of cancer essential medicines in the public sector was slightly lower than in the private sector. In general, prices in Mexico were lower than international reference prices and lower than other LATAM countries. Only few medicines were considered affordable in both the public and private sectors; affordability of medicines in the public sector (14% of medicines) was slightly better than in the private sector (10% of medicines).

### Availability of cancer medicines

The average availability of cancer medicines was lower in the public sector (61.2%) than in the private sector (67.45%), with high variability across individual medicines. The availability of SPS medicines was slightly higher (70.2%), especially for those for breast cancer and leukemia. Greater availability reflects an increased government investment in these two types of cancer treatments. Access to colorectal cancer medicines was lower than breast cancer and leukemia medicines; barriers to access essential medicines for colorectal cancer have been reported [22], despite the fact that colorectal cancer is covered by SPS. Renal cancer medicines for adult patients had the lowest availability of all, likely because they are not covered by SPS and therefore deprioritized [38, 39].

Overall, the availability of cancer medicines falls below the World Health Organization’s (WHO) target of 80% for essential medicines to treat major NCDs (which includes cancer) [40–42]. Thus, mechanisms to improve the availability of cancer medicines in Mexico are warranted. Low availability in the private sector could be explained by the fact that specialized medicines are marketed in selected pharmacy chains only. Therefore, patients have a limited choice of pharmacies, restricted to some states and mostly in urban areas.

The availability of cancer medicines in Mexico’s public sector is higher than other LMIC countries from which data were available. For example, studies in Tanzania and Pakistan [12, 13] reported 50% availability of cancer medicines in the public sector. In Pakistan, the availability of cancer medicines in the private sector was higher than in the public sector, which is a common trend in LMIC [13]. However, private pharmacies in Pakistan focus more on stocking originator brand cancer medicines, while in Mexico private pharmacies have a better availability of generic cancer medicines [13].

### Affordability of cancer medicines

Most cancer medicines are unaffordable for patients, in the private and in three public hospitals where patients paid for cancer medicines. Based on our own definition, only 7 out of 49 cancer medicines were affordable [13, 32]. In addition, most cancer treatment regimens require more than one medicine, making the treatment as a whole even less affordable, often leading to catastrophic expenditures and poverty [6, 43, 44]. The studies from Tanzania and Pakistan also reported that cancer medicines required more than one working day to pay for 1 day of treatment, therefore considered unaffordable to most patients [12, 13]. These results, including Mexico, confirm the LMIC’s general picture of unaffordability of essential cancer medicines. Therefore, more aggressive pricing policies are needed to disrupt this ongoing problem.

We also found that 18 out of 21 of the public hospitals we surveyed reported no additional charges for patients without SPS coverage, as a mechanism to guarantee access to treatment without incurring in health expenditure. For those hospitals that do charge uninsured patients for treatment, these patients must make out-of-pocket payments for (mostly) unaffordable medicines and/or turn to charity organizations [23, 45].

### Procurement prices and international price comparison

We found that, in general, prices of cancer medicines in the public sector in Mexico were lower than other LATAM countries and international reference prices. The Mexican government has contained procurement prices better in the public sector than other countries in the region, through pooled procurement, price negotiations and using reference pricing for SPS medicines [46–49]. For SPS medicines, most public procurement prices were under the SPS reference prices [47, 50], and overall about 20% lower than (MSH) international reference prices. Still, additional efforts are needed to further reduce and monitor prices; in particular for those that are more than twice the reference prices (e.g. docetaxel).

Overall, medicine prices in Mexico’s private sector were lower than retail prices in the regional countries. Yet, current prices, especially for patented medicines, remain unaffordable, warranting the development of comprehensive price regulations schemes, which has not been properly introduced in the country yet [46, 51]. High prices of patented cancer medicines seem common in the region, as other LATAM countries, such as Argentina, Chile, Uruguay, Paraguay and Brazil have reported unaffordable prices as well [52]. Such high prices in the private sector usually lead to catastrophic expenditure, in particular for low-income patients who were unable to get their medicines in the public sector [6]. Besides wider national health insurance coverage for public sector medicines, additional pricing policies are necessary to improve access to more affordable cancer medicines in the private sector.

### Strengths and limitations

To our knowledge, this is the first study on the availability, prices, and affordability of cancer medicines using an adapted form of the WHO/HAI methodology. This study collected data from a representative sample of public hospitals and private pharmacies in eight states. Future research should also consider assessing the availability, affordability, and prices of cancer medicines in other insurance schemes (social health insurance institutions) and other geographic regions of Mexico.

This study has some limitations. At the time of data collection, some medicines were reported as “just became out-of-stock”. Thus, our availability data may underestimate the actual availability of cancer medicines on a regular basis [26]. At one surveyed hospital, some medicine prices were restricted, as this information pertained to the state’s MoH database. In addition, our calculations of patient affordability account for single medicine for 1 day of treatment, which may underestimate the affordability of the treatment as a whole. Furthermore, our affordability assessment in the public sector is limited to the only 3 hospitals that reported patient prices for cancer medicines, which may understate our results on medicine’s affordability in the public sector. However, the current results show that medicines are still unaffordable to patients that receive care at those institutions and pay out of pocket for medicines without coverage.

We only surveyed pharmacies in the private sector because we did not obtain approval from private hospitals to conduct our research. Private hospitals providing cancer care provide chemotherapy at their facilities and care for approximately 19% of cancer cases [53]. Hence, our results do not fully represent the availability, prices, and affordability of cancer medicines in the private sector as a whole. However, the private sector hospitals and clinics represent approximately 2% [54] of the pharmaceutical market. Thus, omitting these data is not likely to have resulted in significant bias in our observations. Additional research is necessary to describe the availability and affordability of cancer medicines in this private subsector.

### Practical implications

Medicines in the public sector covered by SPS were the most available, especially for breast cancer and leukemia. These results reflect the additional investment by the government to improve health care access to priority diseases. We recommend periodically revising and updating the SPS’s protocols for “resource appropriate strategies” [2] that guarantee the best level of care with the most efficacious and cost-effective medicines, including innovative medicines. We also recommend the expansion of SPS coverage to improve access to treatment to all types of cancer.

Overall, the Mexican government has kept prices of cancer medicines in the public sector below prices from other LATAM countries. Yet, most medicines remain unaffordable for patients – particularly for innovative medicines under patent. A comprehensive assessment of the government’s budget allocations and the complete calculation of costs of cancer care (i.e. pharmaceutical and non-pharmaceutical) are required.

The high prices and low affordability of cancer medicines in the private sector reflect a lack of pricing policies and pharmaceutical market regulation. Price monitoring, prices transparency for single-source products, and compulsory licensing when all other measured fail to yield affordable medicines, should be implemented to increase affordability for payers (patients and the health system). Mexico should also consider the full range of pricing policies [55] for medicines in the public and private sectors to assure the provision of affordable medicines for all patients.

Further research is needed to assess the affordability of medicines and comprehensive cancer treatments, both from the patient’s and the health system’s perspective. Continuous monitoring of prices and availability of cancer medicines is necessary to monitor their impact on health expenditure and access to cancer care.

## Conclusions

The availability of cancer medicines in public hospitals and private pharmacies in Mexico need to improve in order to reach WHO’s target of 80% availability. The SPS should be used as a public mechanism to ensure appropriate and timely access to cancer medicines. Although prices in the public sector were lower than in other countries of the region, most cancer medicines continue to be unaffordable to patients in Mexico. Comprehensive pricing policies are needed to improve the affordability of cancer medicines in the public and private sectors.

## Supplementary information

**Additional file 1.** Medicines of study and their characteristics. Characteristics of selected medicines of study following inclusion criteria.

## Abbreviations

CG: Clinical Guidelines
FPGC: Fund against catastrophic expenditures
INCAN: National Institute of Cancerology (Instituto Nacional de Cancerología)
inj: injectable
INSP: National Institute of Public Health (Instituto Nacional de Salud Pública)
IU: International unit
LATAM: Latin America
LMIC: Low- and middle-income countries
LPG: Lowest Price Generic
Mex$: Mexican Peso
mg: milligram
MIC: Middle-income country
ml: milliliter
MoH: Ministry of Health
MPR: Median price ratio
MSH: Management sciences for health
NCD: Non-communicable diseases
NCG: National clinical guidelines
OB: Originator brand
OECD: Organization for economic co-operation and development
PAHO: Pan-American Health Organization
SPS: People’s Health Insurance (Seguro Popular de Salud)
tab: tablet
WHO: World Health Organization
