# Supplementary material for: Availability, prices, and affordability of selected essential cancer medicines in a middle-income country – the case of Mexico
Source: BMC Health Serv Res. 2020 May 14;20:424. doi: 10.1186/s12913-020-05167-9 (PMC7222474; doi:10.1186/s12913-020-05167-9)
Supplement: Supplementary file 1 — Additional file 1. Medicines of study and their characteristics. Characteristics of selected medicines of study following inclusion criteria. [file 12913_2020_5167_MOESM1_ESM.docx]

**Appendix 1. Medicines of study and their characteristics**

| No. | Medicine | Disease  Treatment | Patent Status | Covered by SPS | Included in NCG | Included INCAN CG |
| --- | --- | --- | --- | --- | --- | --- |
| 1 | Anastrozole 1mg tab | Breast cancer | Off | Yes | Yes | Yes |
| 2 | Bevacizumab 100mg inj | Colorectal cancer | On | Yes | Yes | Yes |
| 3 | Bevacizumab 400mg inj | Colorectal cancer | On | Yes | Yes | Yes |
| 4 | Capecitabine 500mg tab | Colorectal cancer | Off | Yes | Yes | Yes |
| 5 | Carboplatin 150mg inj | Renal cancer | Off | Yes | Yes | No |
| 6 | Cetuximab 5mg/ml inj | Colorectal cancer | On | Yes | No | Yes |
| 7 | Cyclophosphamide 200mg inj | Renal Cancer | Off | Yes | Yes | Yes |
| 8 | Cyclophosphamide 500mg inj | Renal Cancer | Off | Yes | Yes | Yes |
| 9 | Cytarabine 500mg inj | Leukemia | Off | Yes | Yes | Yes |
| 10 | Dasatinib 50mg tab | Leukemia | On | Yes | Yes | No |
| 11 | Daunorubicin 20mg inj | Leukemia | Off | Yes | Yes | Yes |
| 12 | Docetaxel 20mg/1ml inj | Breast cancer | Off | Yes | Yes | No |
| 13 | Docetaxel 80mg/4ml inj | Breast cancer | Off | Yes | Yes | No |
| 14 | Doxorubicin 10mg inj | Breast cancer | Off | Yes | Yes | Yes |
| 15 | Doxorubicin 50mg inj | Breast cancer | Off | Yes | Yes | Yes |
| 16 | Epirubicin 10mg/5ml inj | Breast cancer | Off | Yes | Yes | No |
| 17 | Epirubicin 50mg/25ml inj | Breast cancer | Off | Yes | Yes | No |
| 18 | Etoposide 20mg/ml inj | Renal cancer | Off | Yes | Yes | No |
| 19 | Everolimus 10mg tab | Renal cancer | On | No | Yes | Yes |
| 20 | Everolimus 5mg tab | Renal cancer | On | No | Yes | Yes |
| 21 | Exemestane 25mg tab | Breast cancer | Off | Yes | Yes | Yes |
| 22 | Fluorouracil 250mg inj | Colorectal cancer | Off | Yes | Yes | Yes |
| 23 | Folinic Acid 50mg/4ml inj | Colorectal cancer | Off | Yes | No | Yes |
| 24 | Folinic Acid 15mg tab | Colorectal cancer | Off | Yes | No | Yes |
| 25 | Gemcitabine 1g inj | Renal cancer | Off | No | Yes | Yes |
| 26 | Ifosfamide 1g inj | Renal cancer | Off | Yes | Yes | No |
| 27 | Imatinib 100mg tab | Leukemia | On | Yes | Yes | Yes |
| 28 | Imatinib 400mg tab | Leukemia | On | Yes | Yes | Yes |
| 29 | Irinotecan 20mg/ml inj | Colorectal cancer | Off | Yes | No | Yes |
| 30 | L-Asparaginase 10000IU inj | Leukemia | On | Yes | Yes | No |
| 31 | Letrozole 2.5mg tab | Breast cancer | Off | Yes | Yes | Yes |
| 32 | Mercaptopurine 50mg tab | Leukemia | On | Yes | Yes | No |
| 33 | Methotrexate 2.5mg tab | Leukemia | Off | Yes | Yes | Yes |
| 34 | Methotrexate 500mg inj | Leukemia | Off | Yes | Yes | Yes |
| 35 | Methotrexate 50mg inj | Leukemia | Off | Yes | Yes | Yes |
| 36 | Nilotinib 200mg tab | Leukemia | On | No | No | No |
| 37 | Oxaliplatin 100mg/20ml inj | Colorectal cancer | Off | Yes | No | Yes |
| 38 | Oxaliplatin 50mg/10ml inj | Colorectal cancer | Off | Yes | No | Yes |
| 39 | Paclitaxel 6mg/ml inj | Breast cancer | Off | Yes | Yes | No |
| 40 | Panitimumab 20mg/ml inj | Colorectal cancer | On | No | No | Yes |
| 41 | Pazopanib 200mg tab | Renal cancer | On | No | Yes | Yes |
| 42 | Pazopanib 400mg tab | Renal cancer | On | No | Yes | Yes |
| 43 | Rituximab 100mg/10ml inj | Leukemia | On | Yes | Yes | No |
| 44 | Rituximab 500mg/50ml inj | Leukemia | On | Yes | Yes | No |
| 45 | Sorafenib 200mg tab | Renal cancer | On | No | No | Yes |
| 46 | Sunitinib 12.5mg tab | Renal cancer | On | No | Yes | Yes |
| 47 | Tamoxifen 20mg tab | Breast cancer | Off | Yes | Yes | Yes |
| 48 | Trastuzumab 440mg inj | Breast cancer | On | Yes | Yes | Yes |
| 49 | Vincristine 1mg inj | Renal cancer | Off | Yes | Yes | Yes |

tab= tablet; inj= injectable; mg= milligrams; ml= milliliters; IU= international unit; SPS = People’s Health Insurance (Seguro Popular de Salud); NCG = National Clinical Guidelines; INCAN = National Institute of Cancerology; CG = Clinical guidelines
